# Molecular docking analysis of compounds from Lycopersicon esculentum with the insulin receptor to combat type 2 diabetes

**DOI:** 10.6026/97320630016748

**Published:** 2020-10-31

**Authors:** Anitha Roy, Ponnulakshmi Rajagopal, Lakshmi Thangavelu

**Affiliations:** 1 Department of Pharmacology, Saveetha Dental College and Hospital, Saveetha Institute of Medical and Technical Sciences, Chennai, Tamil Nadu, India; 2 Central Research Laboratory, Meenakshi Academy of Higher Education and Research (Deemed to be Universiy) Chennai-600 078, India

**Keywords:** Diabetes, Lycopersicon esculentum, molecular docking

## Abstract

It is known that tomato (Lycopersicon esculentum) contains bioactive compounds to combat type-2 diabetes. Therefore, it is of interest to document data from the molecular docking analysis of compounds from Lycopersicon esculentum with the insulin receptors to combat type-2 diabetes. We report the binding features of cinnamic acid, chlorogenic acid, gallic acid & glucoside with insulin receptors for further consideration.

## Background

Type-2 diabetes is one of the greatest global health emergencies of the twenty-first century. Insulin resistance is believed to be the key event contributing to the progression of the disease in type-2 diabetes. Insulin resistance results in reduced glucose absorption by peripheral tissues, as well as impairment in lipid homeostasis. Several tissues have a crucial role to play in the development of insulin resistance and type-2 diabetes. Insulin Receptor (IR) is a tetrameric protein composed of two extracellular alpha subunits and two transmembrane beta subunits [1]. The binding of insulin to the alpha subunit of IR induces conformational changes in the receptor leading to the activation of the tyrosine kinase beta subunit. Activated IR also has capacity to auto phosphorylate and phosphorylate intracellular substrates which are necessary for the initiation of additional cellular responses to insulin [2-4]. Such activities result in activation of downstream signaling molecules that participate in the insulin-signaling pathway [5]. Insulin signaling, including activation of IR tyrosine kinase, was disrupted in most patients with diabetes mellitus. This insulin resistance contributes to hyperglycemia as well as other metabolic disorders of the disease [6]. Therefore, compounds that increase the function of the insulin receptor tyrosine kinase may be useful in the treatment of diabetes mellitus.

It is known that tomato (Lycopersicon esculentum) contains bioactive compounds to combat type-2 diabetes [7,8]. Therefore, it is of interest to document data from the molecular docking analysis of compounds from Lycopersicon esculentum with the insulin receptors to combat type-2 diabetes. We report the binding features of cinnamicacid, chlorogenicacid, gallicacid & glucoside with insulin receptors for further consideration.

## Materials & Methods:

### Protein target:

**Figure 1:**
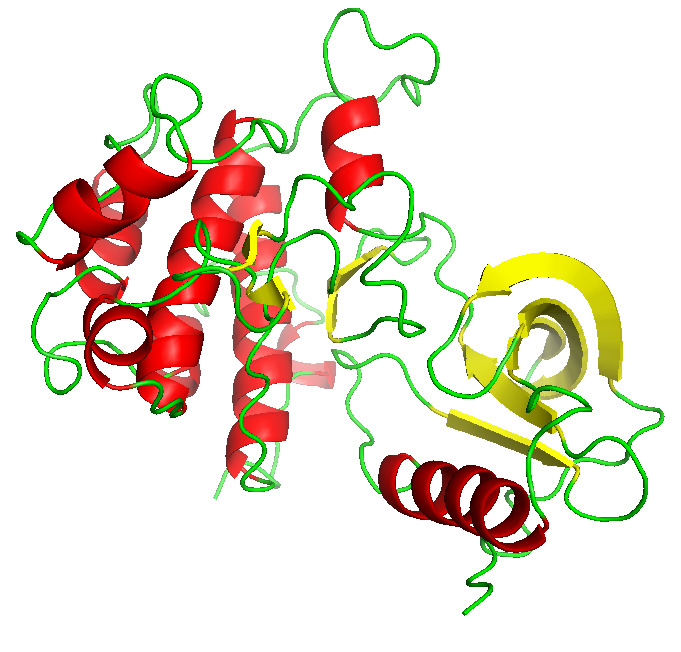
Structure of the insulin receptor

The high-resolution structure of Insulin receptor was downloaded from PDB (PDB Id: 1IRK) [9] (Figure 1).

### Ligand preparation:

12 compounds identified from the tomato plant were selected from the literature. The structures of these compounds have been retrieved from the PubChem Compound Database in the Spatial Data File (.SDF) file format and converted to the PDB file format using the Online Smile Translator. Energy minimization of ligands was performed using ChemBio 3D Ultra 12.0, based on the method stated.

### Molecular docking:

The Patchdock server was used to determine the interaction between the insulin receptor and the selected compounds. PatchDock identified the top candidate solutions based on the complementary form of soft molecular surfaces. The clustering RMSD was set to 4.0 Å as proposed by the software developer for larger molecules and the complex type was set to default. The PatchDock algorithm divides the Connolly dot surface representation of the molecules into concave, convex and smooth regions. Complementary patches are then coordinated to create the candidate transformations of the docked complex (the candidate transformations are docked complexes of the specified receptor and ligand molecule based on the patchdock theory). The result has been retrieved from the e-mail address given and downloaded [10,11].

## Results and Discussion:

**Figure 2:**
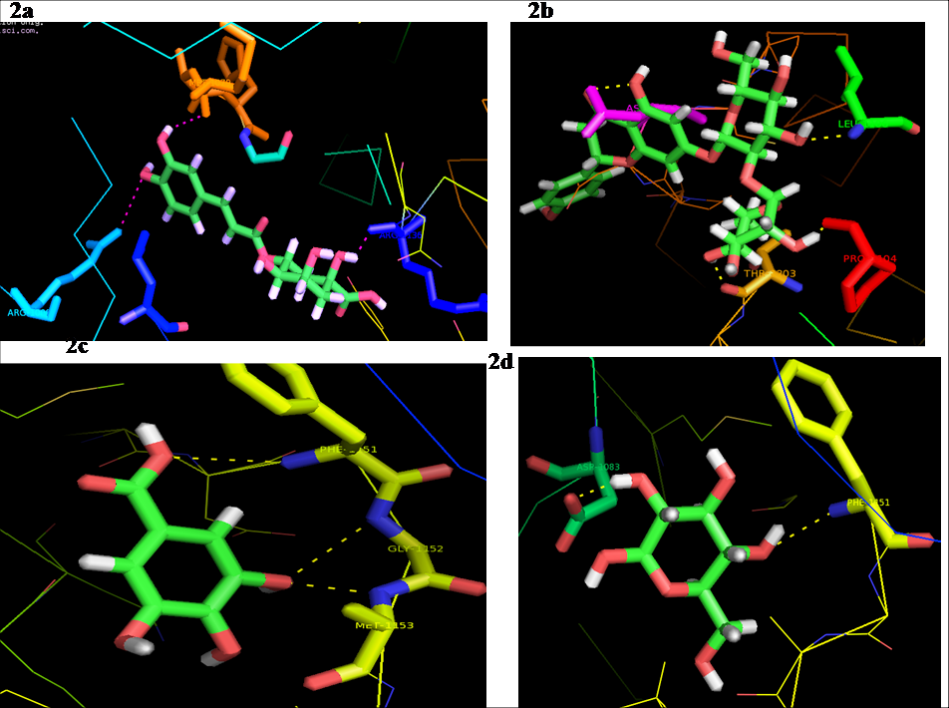
Interaction of the insulin receptor with (a) Chlorogenicacid; (b) Cinnamicacid; (c) Gallicacid; (d) Glucoside

Docking was performed using the PatchDock software between the lead compounds present in Tomato Plants targeting 1IRK to determine the binding efficiency in the form of Atomic Contact Energy (ACE) as shown in Table 1 (see PDF). ACE is a good parameter/indicator to know the docking efficiency of target molecules with lead compounds under the Patch Dock system. All of the selected compounds showed a good interaction with the target insulin protein receptorBased on the score parameters and the H-bond information; we selected the best four compounds. Among them, chlorogenicacid has an ACE value of-141.17 kcal / mol. In order to analysis the docking the H - bond details, docked structure was visualized using PYMOL software. This study showed that the selected four compounds had more than two H-bond interactions with the target insulin receptor protein. Vijayalakshmi et al. 2015 [12] stated that if the H-bond interaction was more, the binding affinity of the ligand was higher. As per Vijayalakshmi et al., selected four compounds indicate that it has very good binding pattern with target protein. Chlorogenicacid formed three H bond interaction with insulin receptor through amino acids residues ARG-1000 MET -1079 & ARG-1116 (Figure 2a).

Cinnamic acid compound interacts with the insulin receptor molecule in a reasonable manner with a strong ACE value of-277.46 kcal / mol, rendering it the second most active product. Four H-bond interactions were established via THR-1203, PRO-1104, LEU-1106 and ASN-1233 (Figure 2b). Gallicacide was well docked with an insulin receptor with an ACE value of-84.94 kcal / mol. Three H-bonds were recognized between IR and Gallicacide. Gallic acid formed H bonds with PHE-1151, GLY-1152 & MET-1153 amino acid residues of IR protein (Figure 2c). Glucoside also demonstrated strong binding to the IR receptor with an ACE value of-54.98 Kcal / mol. The two H-bond interactions with the amino acids ASP-1083 and PHE-1151 were formed (Figure 2).

## Conclusion

We report the binding features of cinnamic acid, chlorogenic acid, gallic acid & glucoside with insulin receptors for further consideration.
